# Semi-automatic thresholding of RV trabeculation improves repeatability and diagnostic value in suspected pulmonary hypertension

**DOI:** 10.3389/fcvm.2022.1037385

**Published:** 2023-01-04

**Authors:** Alistair Macdonald, Mahan Salehi, Samer Alabed, Ahmed Maiter, Ze Ming Goh, Krit Dwivedi, Chris Johns, Marcella Cogliano, Faisal Alandejani, Robin Condliffe, James M. Wild, David G. Kiely, Pankaj Garg, Andrew J. Swift

**Affiliations:** ^1^Department of Infection, Immunity and Cardiovascular Disease, The University of Sheffield, Sheffield, United Kingdom; ^2^Department of Clinical Radiology, Sheffield Teaching Hospitals, Sheffield, United Kingdom; ^3^INSIGNEO, Institute for in Silico Medicine, The University of Sheffield, Sheffield, United Kingdom; ^4^Sheffield Pulmonary Vascular Disease Unit, Royal Hallamshire Hospital, Sheffield, United Kingdom; ^5^Norwich Medical School, University of East Anglia, Norwich, United Kingdom

**Keywords:** cardiac MRI, diagnosis, pulmonary hypertension, trabeculation, right ventricle

## Abstract

**Objectives:**

Right ventricle (RV) mass is an imaging biomarker of mean pulmonary artery pressure (MPAP) and pulmonary vascular resistance (PVR). Some methods of RV mass measurement on cardiac MRI (CMR) exclude RV trabeculation. This study assessed the reproducibility of measurement methods and evaluated whether the inclusion of trabeculation in RV mass affects diagnostic accuracy in suspected pulmonary hypertension (PH).

**Materials and methods:**

Two populations were enrolled prospectively. (i) A total of 144 patients with suspected PH who underwent CMR followed by right heart catheterization (RHC). Total RV mass (including trabeculation) and compacted RV mass (excluding trabeculation) were measured on the end-diastolic CMR images using both semi-automated pixel-intensity-based thresholding and manual contouring techniques. (ii) A total of 15 healthy volunteers and 15 patients with known PH. Interobserver agreement and scan-scan reproducibility were evaluated for RV mass measurements using the semi-automated thresholding and manual contouring techniques.

**Results:**

Total RV mass correlated more strongly with MPAP and PVR (*r* = 0.59 and 0.63) than compacted RV mass (*r* = 0.25 and 0.38). Using a diagnostic threshold of MPAP ≥ 25 mmHg, ROC analysis showed better performance for total RV mass (AUC 0.77 and 0.81) compared to compacted RV mass (AUC 0.61 and 0.66) when both parameters were indexed for LV mass. Semi-automated thresholding was twice as fast as manual contouring (*p* < 0.001).

**Conclusion:**

Using a semi-automated thresholding technique, inclusion of trabecular mass and indexing RV mass for LV mass (ventricular mass index), improves the diagnostic accuracy of CMR measurements in suspected PH.

## Introduction

Pulmonary hypertension (PH) is a life-limiting condition defined by an increase in pulmonary vasculature pressure (1). The established diagnostic criteria was a mean pulmonary artery pressure (MPAP) ≥25 mmHg, however, this has been recently updated to MPAP > 20 mmHg for the definition of PH (1, 2). Right heart catheterization (RHC) is the gold standard method for diagnosing PH (1). However, cardiac MRI (CMR) is an appealing non-invasive alternative that can aid the evaluation of PH by providing information about cardiac morphology and function (3–5).

Pulmonary hypertension involves a persistent increase in afterload for the right ventricle (RV), which undergoes compensatory hypertrophy in an attempt to maintain output. The resulting increase in RV mass is a biomarker for disease severity and can be measured using CMR. The ventricular mass index (VMI = RV mass/LV mass) indicates the degree of RV hypertrophy and has previously been shown to have a high diagnostic accuracy and prognostic value (6–9). However, existing studies have reported different methods for the measurement of ventricular mass, with some having excluded trabeculation and papillary muscles from the final mass measurement (7, 10–13). Trabeculation is defined as the muscular protrusions in the ventricles (14). Furthermore, while some studies suggest that a semi-automatic pixel intensity-based tool is highly reproducible (15, 16) and that manual contouring of trabeculation is poorly reproducible (17, 18), the only direct comparison of these approaches showed that manual contouring was the most reproducible in patients with PH (19, 20).

This study aimed to compare the reproducibility of RV mass measurements with the inclusion or exclusion of trabeculation and to evaluate whether their inclusion in RV mass measurement improves diagnostic accuracy in patients with suspected PH.

## Materials and methods

### Patient selection

**FIGURE 1:**
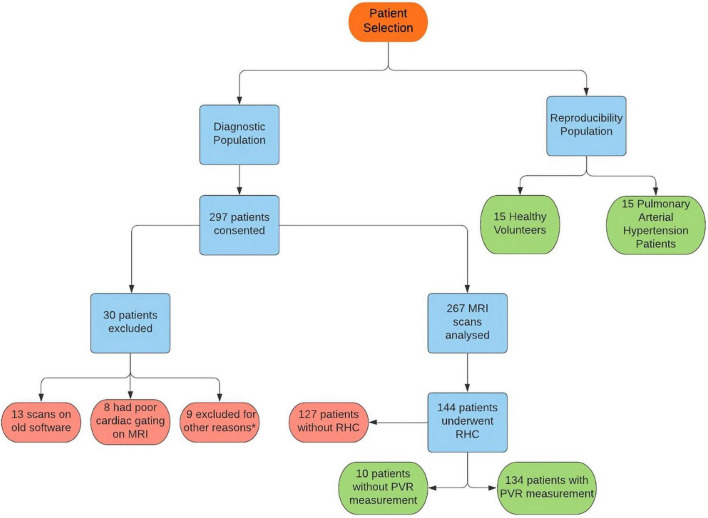
Flowchart showing patient selection for the study. *Six patients were found to have duplicate consent, one patient withdrew due to claustrophobia, one scan could not be found for analysis and one result was excluded as image analysis failed.

Participants were recruited prospectively for two assessments (Figure 1). (i) Diagnostic assessment: consecutive patients with suspected PH when attending for CMR between July 2017 and October 2018 were prospectively identified from the ASPIRE Registry (21) (Figure 1). (ii) Reproducibility assessment: a sample of 30 subjects including 15 patients with incident PAH and 15 healthy volunteers were selected from the RESPIRE study from September 2015 to September 2018 (22) (ClinicalTrials.gov Identifier: NCT03841344). Diagnostic (REC 17/YH/0016) and reproducibility (REC 15/YH/0269) studies received ethics approval from the local Ethics Committee and Institutional Review Board. All patients gave written informed consent.

### Right heart catheterization

A total of 144 of the 267 patients recruited in the study underwent RHC within 2 days of their CMR to obtain MPAP, pulmonary vascular resistance (PVR) and other values. The RHC was performed at the PH referral center by a PH consultant physician (Supplementary Digital Content 1). PH was defined as an MPAP of ≥25 mmHg and MPAP > 20 (1, 2).

### CMR acquisition

Scans were performed at 1.5T on a GE HDx whole body scanner (GE Healthcare, Milwaukee, WI, USA; *n* = 20), or a Phillips Ingenia at 3T (Philips Healthcare; *n* = 10). Short-axis images were captured using a multi-slice balanced steady-state free precession (SSFP) sequence. For the reproducibility assessment, patients underwent two scans on the same day on the same scanner in two separate sessions. Full details about the MRI acquisition are provided in Supplementary Digital Content 1.

### CMR analysis

End-diastolic and end-systolic phase CMR images were analyzed using Qmass (Medis, Leiden, Netherlands). In the diagnostic assessment, MRI analysis was performed by two observers (AJS and DC) each with over 12 years of experience in CMR (Supplementary Digital Content 1). The MassK tool on Qmass excluded areas of lower pixel intensity from the blood pool of the right and left ventricles and defined these areas as trabecular mass. The threshold for this was decided by visual satisfaction; no single threshold could be identified due to the variability of signal intensity of blood and myocardium between individual cases. The total mass value included compacted and trabecular mass and was measured at end-diastole. Mass and volume CMR measurements were indexed to body surface area. VMI was calculated by indexing RV mass to LV mass (RV mass/LV mass).

**FIGURE 2:**
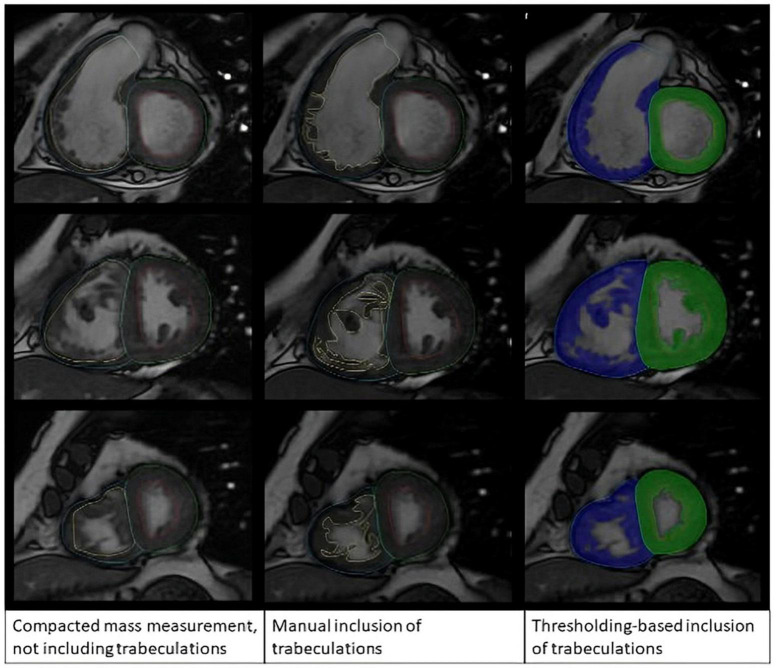
Manual tracing method compared with semi-automatic thresholding method.

In the reproducibility assessment, analyses were performed by two operators trained on Qmass (AS and AMac had 12 and 1 year of experience in CMR, respectively). For the manual RV and LV mass analysis, the myocardium was contoured to include the trabeculation as muscle mass based on visual assessment of low pixel intensity areas in the blood pool (Figure 2). In the threshold technique, the endocardial contour was placed at the compacted endocardial surface, and the MassK threshold tool was selected. RV Myocardial mass including trabeculations was labeled a total mass, while myocardial mass excluding trabeculations was labeled compacted mass. Finally, trabeculation alone was labeled trabecula mass.

For the reproducibility analysis, two observers analyzed the same scan for inter-observer assessment. In addition, for inter-study assessment, one observer analyzed two scans of the same patient performed on the same day. Observers were blinded to each other’s assessment and were timed for contour placement using both methods.

### Statistical analysis

Independent *t*-tests were used to assess group differences. (i) Diagnostic accuracy of RV mass parameters was evaluated with receiver operating characteristic curve (ROC) analysis. Area under the curve (AUC) and the significance of any differences in AUC were tested using the Mann-Whitney test. Correlations between RHC and CMR were calculated using the Pearson correlation coefficients. Paired sample *t*-tests evaluated differences between mean values calculated by each method.

(ii) Intraobserver variability was measured as a component of the repeat scan variability using the Intraclass-correlation-coefficient (ICC) and Bland-Altman analysis (23). Statistical analyses were performed on SPSS version 25.0 (IBM, Chicago, IL, USA). The significance threshold was set at *P* < 0.05.

## Results

### Included cases

A total of 267 patients were recruited (Figure 1); 144 patients underwent RHC within 48 h of CMR and were included in the diagnostic assessment. The population consisted of 41% males and an average age of 63 years old. The underlying diagnosis for the vast majority was either Pulmonary arterial hypertension or chronic thromboembolic PH (47 respectively), whilst a handful had left heart disease (11), PH lung disease (5) or a multifactorial cause (1) (Supplementary Table 1). The reproducibility assessment included 15 healthy volunteers and 15 participants with pulmonary arterial hypertension (PAH).

### Summary statistics for diagnostic population

A total of 114 (79%) patients were diagnosed with PH. Participants with or without PH differed in their invasive hemodynamics and RV CMR metrics apart from end-diastolic volume (*P* = 0.4) and compacted mass (*P* = 0.2) (Supplementary Table 1).

### Correlations

Right ventricle total mass index correlated better with MPAP (*r* = 0.59) and PVR (0.63) than RV compacted mass index (0.25 and 0.38, respectively) (Table 1).

**TABLE 1:** Pearson correlations between RV mass metrics measured on cardiac MRI and right heart catheterization parameters and ROC analysis results using a diagnostic threshold of MPAP ≥ 25 mmHg and MPAP > 20 mmHg.

	Correlations		ROC analysis			
	**MPAP**	**PVR**	**MPAP ≥ 25 mmHg**		**MPAP > 20 mmHg**	
	***R***	***r***	**AUC**	***P*-value**	**AUC**	***P*-value**
Total mass index	0.589	0.626	0.770	<0.001	0.806	<0.001
RV trabecular mass index	0.654	0.632	0.813	<0.001	0.834	<0.001
RV compacted mass index	0.248	0.377	0.606	0.074	0.656	0.005
Percentage trabecular mass	0.544	0.407	0.783	<0.001	0.748	<0.001

ROC, receiver operating characteristic; AUC, area under the curve; MPAP, mean pulmonary artery pressure; PVR, pulmonary vascular resistance.

### ROC analysis

**FIGURE 3:**
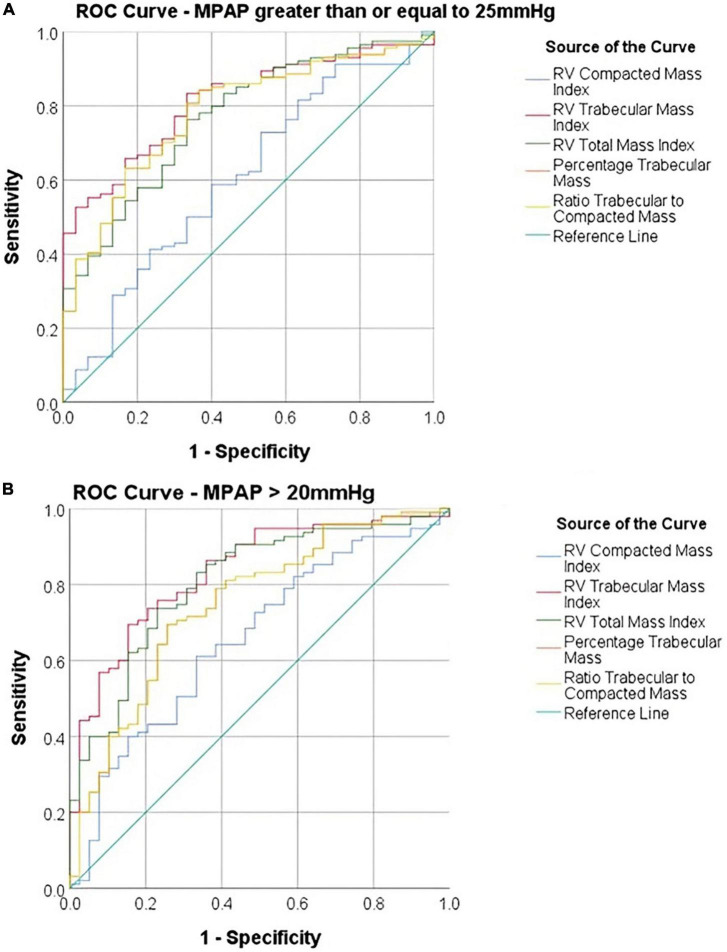
Receiver operating characteristic (ROC) curves comparing diagnostic accuracy of cardiac MRI parameters using **(A)** a diagnostic threshold of MPAP ≥ 25 mmHg and **(B)** a diagnostic threshold of MPAP > 20 mmHg.

The AUC of diagnosing PH with total mass index was 0.77 and for RV, trabecular mass index was 0.81. The compacted mass index was not a significant predictor of PH (AUC = 0.61, *P* = 0.07). Total mass index (*P* = 0.02) and trabecular mass index (*P* = 0.002) were superior predictors to compacted mass (Figure 3 and Table 1). Similarly, using the updated definition, total mass index (AUC = 0.81, *P* = 0.001) and trabecular mass index (AUC = 0.83, *P* = 0.003) were superior to compacted mass (AUC = 0.66, *P* = 0.005).

### Comparison of manual and threshold analysis

Manual contouring produced higher values for end-diastolic volume, end-systolic volume and stroke volume (*P* < 0.001), compared with semi-automatic thresholding. Manual contouring produced lower values for RV total mass, RV trabecular mass and RV percentage trabecular mass (*p* < 0.001) (Supplementary Table 2).

### Speed of methods

The mean time taken to draw the endocardial contour using manual contouring (13 min 2 s, SD = 2 min 49 s) (Supplementary Table 2) was significantly longer (*p* < 0.001) than the mean time taken to gain results using the semiautomatic method (6 min 26 s, SD = 1 min 25 s).

### Interobserver reproducibility

**FIGURE 4:**
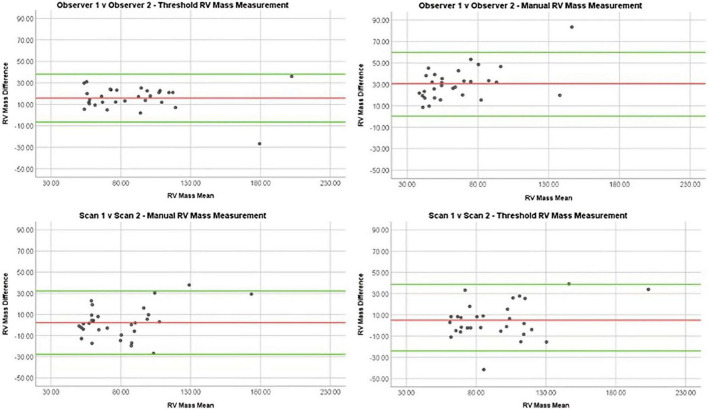
Bland Altman plots demonstrating reproducibility of RV mass measurements using different methods: Manual interobserver reproducibility, threshold interobserver reproducibility, manual repeat scan reproducibility, and threshold repeat scan reproducibility.

The semi-automatic thresholding was more reproducible for all mass measurements. Semi-automatic thresholding also led to less variation for RVSV with ICC of 0.86 in comparison to 0.85 for manual contouring. Similarly, RVEF showed less variation with ICC of 0.97 for the semi-automatic thresholding compared to 0.83 for the manual method. Manual contouring had marginally higher intraobserver reproducibility for RVEDV and RVSEV with ICC of 0.94 and 0.95, respectively when compared to thresholding values of 0.92 and 0.93 (Table 2). Manual contouring was more reproducible for all volumes in patients with PH, however; conversely, mass measurements were more reproducible using the automatic method. Bland-Altman analysis showed that the difference in mass measurement values between our two observers was reduced using the semi-automatic thresholding method (Figure 4). A second sub-analysis (Supplementary Table 3, field strength sub-analysis) showed that a 1.5T scanner using the threshold method had the greatest reproducibility for RV total mass (thresholding 1.5T = 0.87, thresholding 3T = 0.86, manual 1.5T = 0.47, manual 3T = 0.65) and RV trabecular mass (threshold 1.5T = 0.92, threshold 3T = 0.74, manual 1.5T = 0.58, manual 3T = 0.71) as well as RVEDV, RVESV, and RVSV.

**TABLE 2:** Intraclass correlation results comparing interobserver and repeat scan reproducibility for methods 1 (manual tracing) and 2 (semiautomatic pixel-intensity based thresholding), in a mixed population of healthy volunteers and PAH patients, and for PAH patients only.

Variable	Interobserver reproducibility		Repeat scan reproducibility	
	**Method 1 ICC**	**Method 2**	**Method 1**	**Method 2**
ED volume	0.935	0.923	0.946	0.942
ES volume	0.946	0.931	0.960	0.969
Stroke volume	0.851	0.861	0.679	0.778
Ejection fraction	0.833	0.871	0.790	0.869
Total mass	0.527	0.864	0.859	0.849
Compacted mass	0.348	0.348	0.749	0.749
Trabecular mass	0.616	0.847	0.839	0.803
**PH patients only**				
ED volume	0.939	0.925	0.955	0.954
ES volume	0.956	0.930	0.968	0.978
Stroke volume	0.882	0.874	0.685	0.777
Ejection fraction	0.897	0.943	0.811	0.887
Total mass	0.521	0.875	0.797	0.837
Compacted mass	0.328	0.328	0.682	0.682
Trabecular mass	0.573	0.733	0.772	0.852
**Healthy volunteers**				
ED volume	0.904	0.914	0.904	0.905
ES volume	0.854	0.885	0.887	0.902
Stroke volume	0.817	0.854	0.697	0.784
Ejection fraction	0.684	0.658	0.658	0.738
Total mass	0.173	0.556	0.773	0.557
Compacted mass	0.138	0.138	0.727	0.727
Trabecular mass	0.333	0.919	0.751	0.305

### Repeat scan reproducibility

On comparison of MRI parameters between scan 1 and 2, little difference was seen between ICCs of manual contouring and semi-automatic thresholding in RVEDV (0.95 vs. 0.94), RVESV (0.96 vs. 0.97), and RV compacted mass (0.68 vs. 0.68) (Table 2). However, ejection fraction and stroke volume were more reproducible using semi-automatic thresholding (0.87 vs. 0.79 and 0.78 vs. 0.68, respectively), whilst trabecular mass measurement was superior using manual contouring (0.84 vs. 0.80). Compacted mass was moderately reproducible on repeat scans (0.74 for both methods). In the PH group sub-analysis, the thresholding method was more reproducible for measuring RVESV (0.98 vs. 0.97), RVSV (0.78 vs. 0.69), RVEF (0.89 vs. 0.81), RV total mass (0.84 vs. 0.80), and RV trabecular mass (0.85 vs. 0.77). On Bland-Altman analysis (Figure 4), manual and thresholding methods showed mean differences close to zero between scans, with values of 2 and 5 g, respectively. However, both showed wide limits of agreement, with a standard deviation of 15.29 g for the manual method and 17.22 g for the threshold method. Field strength sub-analysis (Supplementary Table 3) showed using a 1.5T scanner and using the thresholding technique to be the most reproducible method for RV total mass (threshold 1.5T = 0.92, threshold 3T = 0.67, manual 1.5T = 0.90, manual 3T = 0.75) and RV trabecular mass (threshold 1.5T = 0.93, threshold 3T = 0.46, manual 1.5T = 0.87, manual 3T = 0.69). Compacted mass was more reproducible using 3T field strength (1.5T = 0.73, 3T = 0.80).

## Discussion

Measurement of RV mass on CMR is useful when evaluating PH, but there is no existing consensus on whether to include trabeculation within the RV mass (7–13). This study included 144 patients with a mean age of 61 years with both CMR and RHC information. We have demonstrated semi-automatic contouring is a reliable method for improving the diagnostic accuracy of PH by including the trabeculation.

### Diagnostic accuracy

We postulate that trabecular mass is an important component of RV hypertrophy related to the increased afterload on the RV. Our data suggest that measurements of compacted mass alone have a weaker correlation with invasively measured pulmonary artery pressure. Given that a trabecular mass is a large component of RV mass, and there is a variable degree of RV trabeculation in normative populations, it is important that RV trabecular mass is considered for RV mass measurement. This study supports the measurement of RV trabeculation by semi-automated pixel intensity thresholding, as this was more reliable than the manual approach. Increased trabeculation mass in the RV has been shown to be a measurable non-invasive marker of PH with association to disease severity (24–27). Van de Veerdonk et al. (20) showed increased correlations with hemodynamics when trabeculations were manually included in RV mass measurement. Our study demonstrated this effect in a large diagnostic population of patients with suspected PH using semi-automatic thresholding to include trabeculations. However, further work to determine the incremental value of MRI to diagnose PH beyond the existing strategy with Echocardiography is recommended. Especially whether MRI metrics of RV mass/trabecular mass or septal deviation increase the diagnostic yield over Echocardiography for patients identified to be at low or intermediate risk.

### Reproducibility of RV measurement

In our key parameters of RV trabecular mass and RV total mass, the semi-automatic thresholding method was more reproducible between observers and scans. Bradlow et al. (19) previously concluded that manual contouring was more reproducible than semi-automatic thresholding. However, our study had a larger population, included healthy controls and used alternative thresholding software. Other studies have demonstrated poor reproducibility of manual contouring in the RV (17) and LV (18).

Studies using Qmass software have shown high intraclass correlations for the semi-automatic method when measuring the RV, which are superior when including trabeculation instead of measuring compacted mass only (17, 18). Of note, the semi-automatic method is not considerably affected by observer experience (28). Studies using thresholding have shown high correlations between RV mass on autopsy and in SSFP MRI sequences when trabeculations were included (29). As expected, the threshold method was faster than manual contouring (17, 20).

### Benefits over previous publications

To our knowledge, this was the first study to investigate the diagnostic performance and reproducibility of manual and semi-automated methods using a prospective cohort of suspected PH patients. While Bradlow et al. (19) also examined repeat scan variability and interobserver variability, our study has included a larger population of suspected PH patients alongside healthy controls. Further work is needed to assess the prognostic value of MRI derived measures of compacted and trabecular myocardial mass in larger cohorts of patients with pulmonary arterial hypertension. Whether prognostic stratification by mass volume phenotypes (9) e.g., low and high-volume mass can be improved by the inclusion or exclusion of trabeculations warrants further investigation.

### Limitations

Determining the border between the RV and right atrium is challenging due to the lack of clear basal RV landmarks, which may have affected CMR values. Our sub-analysis showed that RV total and trabecular mass measurements were more reproducible when 1.5T field strength is used; this could be due to the fact that some 3T scans contained flow artifact that made delineation of the cavity difficult and may account for the difference in mass measurement reproducibility demonstrated between 1.5T and 3T CMR in this study. This study utilized software from a single commercial vendor and software-specific differences in measurements could exist. Finally, although a large prospective cohort was used, future studies could assess the generalizability of these findings using a multicenter population.

## Conclusion

Inclusion of trabeculation improves the diagnostic accuracy of RV mass measurements on CMR in patients with suspected PH, using a semi-automated pixel-intensity based thresholding technique. This study showed that CMR measurements used in the assessment of suspected PH are highly reproducible and highlights the diagnostic utility of CMR in suspected PH.

## Data availability statement

The raw data supporting the conclusions of this article will be made available by the authors, without undue reservation.

## Ethics statement

The studies involving human participants were reviewed and approved by the Local Ethics Committee and Institutional Review Board at the University of Sheffield. The patients/participants provided their written informed consent to participate in this study.

## Author contributions

AlM contributed to the data collection and analysis, visualization of results with figures and tables, writing of the original draft, and reviewing and editing of the manuscript. MS contributed to the data collection, analysis, writing, and reviewing and editing of the manuscript. AhM, ZG, KD, CJ, FA, and RC contributed to the data collection and review and editing of the manuscript. JW and DK contributed to the review and editing of the manuscript. PG and AS contributed to the conceptualization and review and editing of the manuscript, and supervision. All authors edited and approved the final manuscript.
