# Supplementary material for: Semi-automatic thresholding of RV trabeculation improves repeatability and diagnostic value in suspected pulmonary hypertension
Source: Front Cardiovasc Med. 2023 Jan 4;9:1037385. doi: 10.3389/fcvm.2022.1037385 (PMC9845927; doi:10.3389/fcvm.2022.1037385)
Supplement: Supplementary file 1 [file Data_Sheet_1.pdf]

frontiers

Supplementary Material

Supplementary Digital Content 1 - Description of MRI analysis and Right Heart Catheterisation

Reproducibility Study MRI Image Acquisition

MRI scans were carried out on scanners with Field strengths of either 1.5T (GE HDz, GE Healthcare, Milwaukee, WI) or 3T (Ingenia, Phillips Healthcare) so that sub-analyses could be carried out based on Field Strength. 3T scans had an image resolution of 256x256 pixels and 1.5T scans had a resolution of 512x512 pixels. Short axis images were acquired using a multi slice steady state free precession sequence. All scans had either 20 or 30 phases and slice thickness was either 8mm with a 2mm slice gap. The minimum number of slices for each scan was 10. Cine SSFP studies were retrospectively ECG gated and breath held.

Diagnostic Study MRI Image Acquisition

Most scans were performed by a radiographer on a 1.5T Field Strength GE HDz whole body scanner (GE Healthcare, Milwaukee, WI). 3 scans were performed using a Phillips Ingenia 3T scanner (Philips Healthcare), and short axis images were captured. Short axis images were captured using a multi slice balanced steady state free precession (SSFP) sequence. At least 20 phases of the cardiac cycle and at least 10 slices of the heart were captured for each short axis scan. Other views such as a four-chamber view were used to enable more in-depth visualisation of cardiac structures, which aided contour placement by identifying where the right ventricle passes into the right atrium.

MRI Analysis

Epicardial contours were placed on the outside edge of the myocardium and endocardial contours were placed on the inside edge of the compacted myocardium. The compacted mass was defined as a band of low intensity pixels around the high intensity blood pool that did not include any trabecular myocardium. The interventricular septum was defined as left ventricular muscle. Four chamber scans were used to help differentiate the ventricle from atrium. LV contours were drawn in all slices where more than 50% of the LV was surrounded by ventricular muscle. RV contours included a ‘shoulder’ of the ventricle around the right atrium (RA) when seen towards the base of the heart.

Right Heart Catheterisation

Right heart catheterisation was performed at the PH referral centre by a trained PH consultant. The procedure involved using a balloon-tipped 7.5 French thermodilution catheter (Becton-Dickinson, Franklin Lakes, NJ) introduced via a Swann-Ganz catheter, usually via the internal jugular vein.

Supplementary Table 1

Demographic variables and differences between patients with or without pulmonary hypertension, using a diagnosis of MPAP≥25mmHg. CTEPH=Chronic thromboembolic PH, RA=Right atrium, MPAP=Mean pulmonary arterial pressure, PAWP=Pulmonary arterial wedge pressure, PVR=Pulmonary vascular resistance, LV= Left ventricle, RV=Right ventricle, EDV=End diastolic volume, ESV=End systolic volume, SV=Stroke volume, EF=Ejection Fraction.

	MPAP <25<br>No PH	MPAP ≥25<br>PH	All Patients	P value
Age in years – mean (SD)	61 (11)	64 (14)	63 (14)	0.283
Female sex – n (%)	20 (66)	65 (57)	85 (59)	0.339
Diagnosis				
Group 1 Pulmonary arterial hypertension		47	47	
Group 2 PH Left Heart Disease		12	12	
Group 3 PH Lung disease		6	6	
Group 4 CTEPH		47	47	
Group 5 Unclear/Multifactorial		2	2	
WHO Functional Class				
I	1	1	2	
II	9	9	18	
III	19	92	111	
IV	1	12	13	
RA Pressure (mmHg)- median (IQR)	3.5 (5.7)	7 (6.0)	6.0 (5.5)	0.001
MPAP (mmHg)- median (IQR)	19.2 (4.1)	42.0 (16.7)	37.0 (20.2)	<0.001
PAWP (mmHg)- median (IQR)	8.0 (3.7)	10.0 (5.0)	10.0 (6.0)	<0.001
PVR (dyne/sec/cm^5)- median (IQR)	170 (140)	483 (532)	403 (452)	<0.001
LV EDV (ml)- median (IQR)	106 (34)	89 (39)	93 (41)	0.266
LV ESV (ml)- median (IQR)	37 (24)	30 (22)	31 (24)	0.564
LV SV (ml)- median (IQR)	69 (26)	58 (25)	60 (26)	0.239
LV EF (%)- median (IQR)	69 (13)	67 (14)	68 (14)	0.669
LV Mass (g)- median (IQR)	136 (53)	123 (44)	124 (47)	0.512
RV EDV (ml)- median (IQR)	104 (56)	119 (76)	116 (79)	0.401
RV ESV (ml)- median (IQR)	44 (31)	64 (49)	64 (50)	<0.001
RV SV (ml)- median (IQR)	58 (33)	47 (31)	48 (29)	0.027
RV EF (%)- median (IQR)	55 (13)	40 (20)	44 (22)	<0.001
RV total mass (g)- median (IQR)	65 (25)	88 (50)	82 (47)	<0.001
RV trabecular mass (g)- mean (SD)	35 (14)	60 (29)	55 (28)	<0.001
RV compacted mass (g)- mean (SD)	34 (11)	37 (13)	36 (130	0.208
RV trabecular mass percentage (%)- mean (SD)	51 (8)	60 (10)	58 (11)	<0.001
Ratio of trabecular to compacted mass- mean (SD)	1.08 (0.38)	1.67 (0.67)	1.55 (0.67)	<0.001

Supplementary Table 2

Mean and standard deviations for cardiac MRI parameters using the manual contouring (Method 1) and semi-automatic thresholding (Method 2), with independent t test results to compare significant differences between the two methods.

Variable	Method 1	Method 2	P value
Measurement time (mins/secs) – mean (SD)	13m2s (2m49s)	6m26s (1m25s)	<0.001
RV ED Volume (ml)- mean (SD)	155 (46)	135 (42)	<0.001
RV ES Volume (ml)- mean (SD)	78 (36)	66 (34)	<0.001
RV Stroke Volume (ml)- mean (SD)	77 (21)	69 (19)	<0.001
RV Ejection Fraction (%)- mean (SD)	50.8 (10)	52.3 (11)	<0.001
RV Total Mass (g)- mean (SD)	70 (30)	90 (34)	<0.001
RV Trabecular Mass (g)- mean (SD)	29 (17)	49 (23)	<0.001
RV Compacted Mass (g)- mean (SD)	41 (16)	41 (16)	N/A
Percentage Trabecular Mass (%)- mean (SD)	39 (12)	54(11)	<0.001

Supplementary Table 3

Sub-analysis of reproducibility between 1.5T and 3T scans. Method 1 - manual contouring. Method 2 - semi-automatic thresholding

Variable	Interobserver Reproducibility		Repeat Scan Reproducibility	
	Method 1 ICC	Method 2 ICC	Method 1 ICC	Method 2 ICC
**1.5T FIELD STRENGTH**				
ED volume	0.931	0.969	0.929	0.924
ES volume	0.941	0.978	0.960	0.944
Stroke Volume	0.880	0.907	0.739	0.808
Ejection Fraction	0.833	0.858	0.813	0.844
Total Mass	0.471	0.870	0.897	0.919
Compacted Mass	0.254	0.254	0.732	0.732
Trabecular Mass	0.583	0.923	0.868	0.932
**3T FIELD STRENGTH**				
ED volume	0.947	0.870	0.968	0.961
ES volume	0.955	0.900	0.961	0.982
Stroke Volume	0.739	0.720	0.453	0.663
Ejection Fraction	0.836	0.883	0.733	0.894
Total Mass	0.648	0.863	0.755	0.673
Compacted Mass	0.500	0.500	0.801	0.801
Trabecular Mass	0.708	0.742	0.689	0.463
